# Identification of highly cross-reactive immunogens in *Eimeria tenella* sporozoites

**DOI:** 10.1051/parasite/2025071

**Published:** 2025-12-02

**Authors:** Weiyi Chen, Tao Han, Feng Song, Haipeng Zhang, Xiangqin Wang, Haiwei Gong, Liheng Liu

**Affiliations:** 1 Jiangxi Provincial Key Laboratory for Animal Health, College of Animal Science and Technology, Jiangxi Agricultural University Nanchang 330045 Jiangxi Province PR China; 2 Institute of Veterinary Medicine/Research Center of Animal Clinical Medicine, Xinjiang Academy of Animal Science Urumqi 830011 Xinjiang PR China; 3 Jiangxi Institute of Science and Technology Information Nanchang 330046 Jiangxi Province PR China; 4 Jiujiang Lilai Biological Science and Technology Co. LTD, Shacheng Industrial Zone of Jiujiang County Jiujiang 332100 Jiangxi Province PR China; 5 Agriculture and Rural Bureau of Chaisang District Jiujiang 332199 Jiangxi Province PR China

**Keywords:** *Eimeria tenella*, Sporozoite, Immunoproteomic, Common immunogenic protein, MALDI-TOF-MS analysis

## Abstract

Avian coccidiosis significantly impairs intestinal health in chickens and remains a major threat to the poultry industry worldwide. Frequent co-infections with three *Eimeria* species, *i.e.*, *Eimeria tenella*, *E. necatrix*, and *E. maxima*, present challenges for effective vaccine development. Here, we first used two-dimensional gel electrophoresis and silver staining to identify 650 *E. tenella* sporozoite proteins and then detected 18 cross-reactive immunogens based on Western blotting and proteomic analysis. These immunogens were consistently recognized by hyperimmune sera raised against three *Eimeria* species of interest. Bioinformatics analysis revealed that these proteins encompass enzymes, motility-related proteins, nuclear factors, and translation machinery, with amino acid sequence identities ranging from 71.1%–98.8% with *E. necatrix* and 37.9%–87.5% with *E. maxima* homologues. Seven of these proteins show potential non-classical secretion, and four have transmembrane domains. Overall, these findings point to multiple cross-reactive immunogens as potential candidates for multivalent coccidiosis vaccines.

## Abstract (fr)

La coccidiose aviaire altère significativement la santé intestinale des poulets et représente une menace majeure pour l’industrie avicole mondiale. Les co-infections fréquentes par trois espèces d’*Eimeria* (*Eimeria tenella, E. necatrix et E. maxima*) complexifient le développement de vaccins efficaces. Dans cette étude, nous avons d’abord identifié 650 protéines de sporozoïtes d’*E. tenella* par électrophorèse bidimensionnelle et coloration à l’argent, puis détecté 18 immunogènes à réactivité croisée par Western blot et analyse protéomique. Ces immunogènes ont été systématiquement reconnus par des sérums hyperimmuns dirigés contre les trois espèces d’*Eimeria* étudiées. L’analyse bioinformatique a révélé que ces protéines comprennent des enzymes, des protéines impliquées dans la motilité, des facteurs nucléaires et des composants de la machinerie de traduction, avec des identités de séquence en acides aminés allant de 71,1 % à 98,8 % avec les homologues d’*E. necatrix* et de 37,9 % à 87,5 % avec les homologues d’*E. maxima*. Sept de ces protéines présentent un potentiel de sécrétion non classique, et quatre possèdent des domaines transmembranaires. Dans l’ensemble, ces résultats suggèrent que de multiples immunogènes à réactivité croisée pourraient constituer des candidats potentiels pour des vaccins multivalents contre la coccidiose.

**Abbreviations**
2-DE: Two-dimensional gel electrophoresis;
ABC: ATP-binding cassette;
PBS: Phosphate-buffered Saline;
PBST: PBS with Tween-20;
IEF: Isoelectric focusing;
IgY: Immunoglobulin of yolk;
IPG: immobilized pH gradient;
MALDI-TOF-MS/MS: Matrix-assisted laser desorption/ionization-time of flight-mass spectrometry/mass spectrometry;
MS: Mass spectrometry;
PMF: Peptide mass fingerprinting;
PSMD: 26S proteasome non-ATPase regulatory subunit;
SDS-PAGE: Sodium dodecyl sulphate polyacrylamide gel electrophoresis.

## Introduction

As an intestinal parasite in chickens, *Eimeria tenella* invades and rapidly multiplies within host cells to cause severe damage to intestinal mucosa, triggering disease manifestations that are collectively known as avian coccidiosis [7]. Multiple pathogenic *Eimeria* species, including *E. tenella*, *E. necatrix* and *E. maxima*, are prevalent worldwide [2], to the extent that co-infections with multiple *Eimeria* species are actually more common than single-species infection [1, 17]. Thus, developing a vaccine that provides cross-protective immunity against multiple pathogenic *Eimeria* species is desirable.

Currently, anticoccidial drugs and live attenuated vaccines have proved to be effective in preventing coccidiosis, but concerns about drug resistance and drug residues, as well as the potential reversal of vaccine strains to the wild-type (virulent) forms are already widespread in the field [8, 23]. Alternative prevention and control measures, such as DNA vaccines and recombinant subunit protein vaccines, have become the focus of ongoing coccidiosis prevention research [9, 15, 33].

Immunoproteomics has been successfully utilized to screen antigens at different developmental stages of chicken *Eimeria* and other parasites of veterinary importance. For example, Udonsom *et al.* [30] identified specific immunoreactive proteins of *Neospora caninum* sporozoites recognized by sera from cattle infected with various parasites using two-dimensional gel electrophoresis (2-DE) combined with immunoblotting and LC-MS/MS. A total of 20 specific antigen spots corresponding to 14 different antigen proteins were identified among 70 immunogens. Similarly, Qu *et al.* [29] used 2-DE coupled with Western blotting to systematically screen *E*. *necatrix* sporozoite proteins, identifying 98 distinct protein spots exhibiting cross-reactivity with species-specific hyperimmune serum derived from *E*. *necatrix* among a total of 680 protein spots resolved. Liu *et al.* [21] analyzed 620 soluble proteins from *E. acervulina* sporozoites and detected 21 conserved antigens that could be simultaneously recognized by hyperimmune sera raised against three *Eimeria* species. These findings indicate the existence of immunogenic and conserved antigens among different *Eimeria* species and justify a further refinement of feasible vaccine-targets shared by chicken *Eimeria*.

The key to developing an effective recombinant vaccine against chicken *Eimeria* lies not only in the quantity of antigens, but also in the selection of optimal and conserved immunogens that can effectively induce protective immune responses [5, 16]. By our own analyses, it is now evident that nearly two dozen *E. tenella* sporozoite antigens that are recognized by hyperimmune sera against *E. tenella*, *E. necatrix*, and *E. maxima* can serve as a good starting point for the design of a recombinant vaccine targeting all three common *Eimeria* species.

## Materials and methods

### Ethical statement

All animal research protocols for this study were approved by the Animal Ethics Committee of Jiangxi Agricultural University (JXAULL2022-025). All experiments were conducted following explicit guidelines of the Experimental Animal Committee under the Ministry of Agriculture and Rural Affairs in China.

### Parasites

San Huang chicks were kept for 14 days in a coccidia-free environment before oral inoculation with 2 × 10^4^ sporulated *E. tenella* oocysts. From day 6 to day 8 post-infection, fecal samples were collected for harvesting oocysts using a saturated saline flotation method [11]. The oocysts were incubated in 2.5% (w/v) potassium dichromate solution at 29 °C for 96 h before storage at 4 °C. Sporulated oocysts were mechanically disrupted using a tissue homogenizer: an equal volume of glass beads to the oocyst pellet was added, and the mixture was processed at 4 °C with a frequency of 70 Hz (10 s operation followed by 10 s pause, repeated for 35 cycles) until the excystation rate reached ≥ 80%. The resulting sporocysts were purified using 50% Percoll gradient centrifugation and then treated with 10% (v/v) San Huang chicken bile and 0.75% (w/v) trypsin at 41 °C for 1 h to fully release sporozoites. After further centrifugation and filtration through a 1,400-mesh sieve, the sporozoites were stored in liquid nitrogen for subsequent use [3].

### Sporozoite proteins

Following the established Liu *et al.* protocol [21], purified *E. tenella* sporozoites were suspended in cell lysis buffer containing 8M urea. The suspension was subjected to sonication on ice bath to lyse cells and release soluble proteins. After centrifugation at 15,000× rpm for 10 min at 4 °C, the supernatant containing soluble protein was collected and processed with a 2-D clean-up kit. Protein concentration was quantified with a PlusOne^TM^ 2-D Quant Kit (Cytiva, Marlborough, MA, USA).

### Isoelectric focusing (IEF)

IEF was performed as previously described [21, 22]. Briefly, *E*. *tenella* sporozoite proteins were rehydrated in IPG buffer, incubated for 1 h at room temperature, and centrifuged. Then, 200 μg of protein was loaded onto 24 cm non-linear pH 3–10 IPG strips (Cytiva). Electrophoresis was performed using a PROTEAN IEF cell (Bio-Rad, Hercules, CA, USA) with four-step voltage: S1 (0–50 V, 12 h), S2 (50–8,000 V, 4 h), S3 (8,000–10,000 V, 4 h), and S4 (10,000 V, 4 h). The strips were immediately used for 2-DE.

### Sodium dodecyl sulphate polyacrylamide gel electrophoresis (SDS‑PAGE)

Before SDS-PAGE, each gel strip was incubated for 15 min in equilibration buffer I and II respectively, following the previously described method [21]. Subsequently, SDS-PAGE was performed using 12.5% polyacrylamide gels on the Ettan DALTtwelve system (Cytiva). For Western blotting and silver staining, two separate gels were run simultaneously. The electrophoresis procedure was as follows: apply 3 W/gel was for 45 min first, then increase to 15 W/gel until the tracking dye reached the bottom of the gels. The temperature during electrophoresis was maintained at 16 °C. Gel staining was carried out following the method of Zhu *et al.* [38], and the gels were imaged using the ArtixScan 1010 Plus (Microtek International, Inc., Hsinchu, Taiwan).

### Digital imaging analysis

The 2-DE gels were analyzed using ImageMaster^TM^ 2D Platinum Software (Version 5.0, Cytiva) for spot detection, quantification, as well as comparative and statistical analyses [21].

### Immune sera

Using a previously described method [36], 14-day-old San Huang chicks were randomly divided into four groups (*n* = 25), namely, experimental groups infected with *E*. *tenella*, *E*. *necatrix*, *E*. *maxima* and a negative control group without infection. Each group was kept separately in chicken coops to prevent cross-contamination. For the three experimental groups, each bird was infected with 5 × 10^4^ sporulated oocysts of the corresponding *Eimeria* species at two weeks of age, and infections were repeated with 5 × 10^3^ sporulated oocysts every three days (on days 3, 6, 9, and 12) to generate hyperimmune sera, while birds in the negative control group received sham inoculation (with distilled water) only. Five weeks after the last inoculation, blood samples were obtained *via* cardiac puncture, and serum antibody titers were determined by ELISA. Samples with titers greater than 1:1,280 were pooled, aliquoted and stored at −20 °C until use for immunoassays, including Western blot.

### Western blotting

In order to obtain uniform protein blotting, each 2-DE gel was cut into four equal parts for transfer. Resulting proteins were transferred to PVDF membranes (Cytiva) following Qu *et al.* [29], membranes were blocked with 5% skim milk in PBST (PBS, pH 7.4, 0.05% Tween 20) for 2 h at room temperature. Three sera, diluted 1:100 in PBST, were incubated with the membranes at room temperature for 2 h, using serum as a negative control. After washing the membranes three times with PBST, membranes were incubated with goat anti-chicken IgG-HRP (1:2,000; Proteintech Group, Inc., Rosemont, IL, USA) for 2 h at 37 °C. Washed again with PBST for 1 h, membranes were developed with a chemiluminescence kit. Imaging and analysis were done on a ChemiDoc^TM^ XRS + with Image Lab^TM^ software (Bio-Rad) [32].

### MS analysis and database searches

Protein spots were subjected to MS analysis at the experimental center of Nanjing Medical University, using a MALDI-TOF/TOF instrument (Bruker Daltonics, Bremen, Germany). The resulting mass fingerprinting (PMF) data were acquired and analyzed using the Mascot search engine (https://www.matrixscience.com). The parameters for protein retrieval were as follows: 100 ppm mass accuracy, one missed trypsin cleavage site allowed, fixed modifications of carbamidomethyl (C), variable modifications of oxidation (M), 100 parts per million mass accuracy, and MS/MS fragment tolerance set to 0.4 Da. A positive protein search result was determined by a Mascot score of more than 71 (*p* < 0.05), amino acid sequence coverage greater than 15%, and at least 4 matching peptides [31]. Prediction of non-classical secretion pathways, signal peptides, and transmembrane structures for the positively identified proteins detected by MALDI-TOF/TOF relied on three online servers, *i.e.*, SecretomeP-2.0a Server (https://services.healthtech.dtu.dk/services/SecretomeP-2.0/), SignalP-5.0 Server (https://services.healthtech.dtu.dk/services/SignalP-5.0/), and TMHMM Server v. 2.0 (https://services.healthtech.dtu.dk/services/TMHMM-2.0/) were used.

## Results

### Proteins revealed by two-dimensional gel electrophoresis (2-DE)

**Figure 1:**
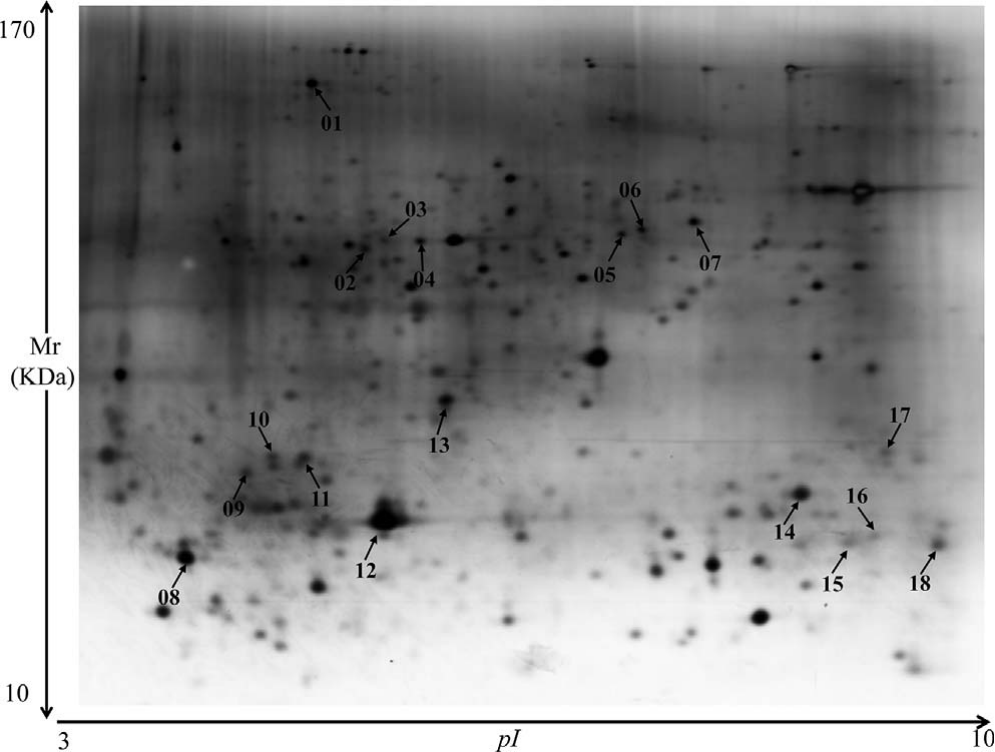
*Eimeria tenella* sporozoites proteins revealed by silver staining of two-dimensional polyacrylamide gel. Approximately 650 spots were detected, primarily between 10–170 kDa and pI 3–10.

In silver-stained 2-DE gels, 650 protein spots were detected in the total *E*. *tenella* sporozoite protein extract, with molecular weights primarily ranging from 10 kDa to 170 kDa (Fig. 1).

### Detection of cross-reactive immunogens by Western blot

**Figure 2:**
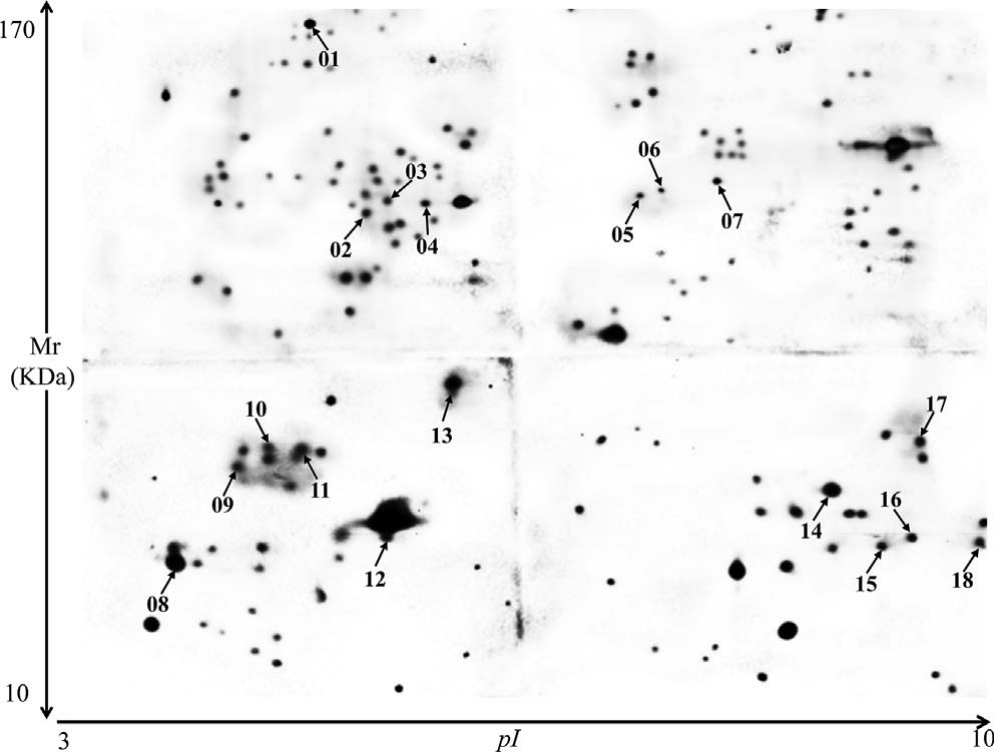
Western blot using *Eimeria tenella* sporozoite proteins and hyperimmune serum raised against *E. tenella*. In all, 151 spots were recognized by anti-*E. tenella* serum, including 18 that were reactive to immune sera against two other *Eimeria* species (Figs. 3–4).

**Figure 3:**
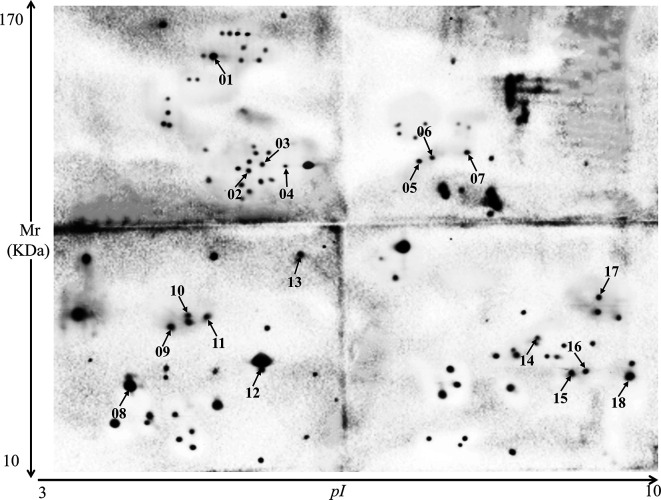
Western blot using *Eimeria tenella* sporozoite proteins and hyperimmune serum raised against *E. maxima*. A total of 103 spots were recognized by anti-*E. maxima* serum, including 18 that were reactive to immune sera against two other *Eimeria* species (Figs. 2 and 4).

**Figure 4:**
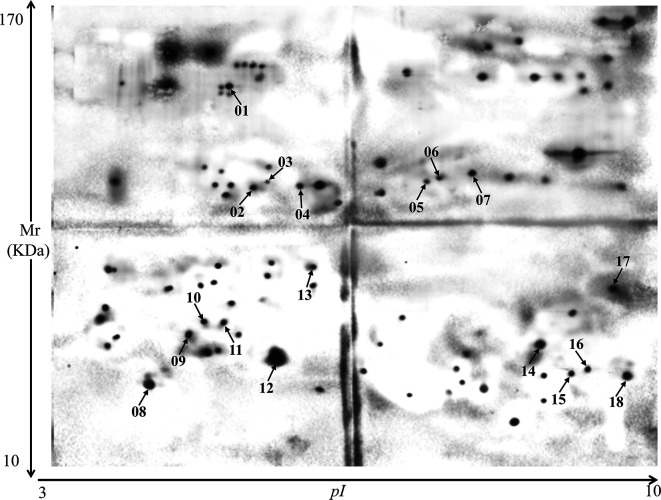
Western blot using *Eimeria tenella* sporozoite proteins and hyperimmune serum raised against *E. necatrix*. A total of 84 spots were recognized by anti-*E. necatrix* serum, including 18 that were reactive to immune sera against two other *Eimeria* species (Figs. 2–3).

In Western blot assays using hyperimmune sera against three different *Eimeria* species, 151 proteins were recognized by anti-*E*. *tenella* sera, 84 by anti-*E*. *necatrix* sera, and 103 by anti-*E*. *maxima* sera. Among these readily identified immunogens, 18 *E*. *tenella* sporozoite proteins were reactive with all three hyperimmune sera (Figs. 2–4).

### Identities of cross-reactive immunogens

By MALDI-TOF/MS analysis, the 18 conserved immunogens in *E. tenella* sporozoites could be classified into five, non-overlapping categories based on enzymatic activities and motor functions (Table 1). In sequence alignment and structural analysis, these *E. tenella* sporozoite proteins showed a broad range of homology with two other related species: from 71.1% to 98.8% with *E. necatrix* and from 37.9% to 87.5% with *E. maxima* (Table 2). Based on BLAST searches, four proteins showed over 80% amino acid sequence identity across all three *Eimeria* species of interest. In terms of functional pathways, using the SecretomeP – 2.0a server, SignalP – 5.0 server, and TMHMM server v2.0 to analyze the non-classical secretory property (SecP), signal peptide (SP), and transmembrane domain (TM), respectively, seven were found to have high SecP scores (>0.6), while four others had transmembrane domains. These proteins almost universally lack signal peptides and are predicted to participate in intercellular signaling (as receptors or signaling molecules) (Table 1).

**Table 1:** Immunoproteomic and mass-spectrometric identification of *Eimeria tenella* sporozoite proteins recognized by hyperimmune sera raised against three *Eimeria* species of interest.

Spot ID^a^	Identified protein grouped by functions	Accession number^b^	No. of matched peptides^c^	Theoretical Mr/pI	Protein score^d^	Protein sequence coverage (%)^e^
**Group 1**	**Proteins of known or predicted enzymatic activities**					
01	ATP-binding cassette protein subfamily B member 2, putative [*Eimeria tenella*]	XP_013228359.1	16/41	147997/5.56	108	14%
02	ATPase, AAA family domain-containing protein, putative, partial [*Eimeria tenella*]	XP_013235934.1	16/32	50654/9.11	204	32%
09	adenylate kinase, putative [*Eimeria tenella*]	XP_013232446.1	16/53	28214/8.59	211	70%
10	glutathione/thioredoxin peroxidase, putative [*Eimeria tenella*]	XP_013231991.1	15/37	27911/9.20	175	53%
11	purine nucleoside phosphorylase, putative [*Eimeria tenella*]	XP_013234769.1	13/37	27907/6.52	155	40%
14	vacuolar ATP synthase subunit d, putative [*Eimeria tenella*]	XP_013233504.1	18/35	27425/9.46	263	79%
**Group 2**	**Protein transport**					
03	vacuolar sorting receptor protein, putative [*Eimeria tenella*]	XP_013229187.1	15/43	61219/6.58	134	31%
12	mitochondrial import inner membrane translocase subunit tim17, putative [*Eimeria tenella*]	XP_013227835.1	15/55	21658/9.38	214	62%
**Group 3**	**Motor-related proteins**					
04	endonuclease/exonuclease/phosphatase domain-containing protein, putative [*Eimeria tenella*]	XP_013229466.1	12/45	51059/7.15	161	31%
05	kinesin motor domain-containing protein, putative [*Eimeria tenella*]	XP_013233623.1	20/43	61229/5.95	179	40%
07	Peptidyl-prolyl cis-trans isomerase d-like protein, related [*Eimeria tenella*]	XP_013230117.1	13/44	63615/5.58	100	27%
08	ATP-dependent metalloprotease ftsh, putative (Fragment) [*Eimeria tenella*]	XP_013231415.1	9/41	14431/5.42	119	34%
16	26S proteasome non-ATPase regulatory subunit, putative, partial [*Eimeria tenella*]	XP_013229897.1	12/49	17586/7.57	151	52%
**Group 4**	**Proteins of nuclear location & function**					
06	meiosis-specific nuclear structural protein 1, putative [*Eimeria tenella*]	XP_013229795.1	18/34	60929/7.66	146	35%
13	Transcription factor IIH component, related, partial [*Eimeria tenella*]	XP_013234425.1	15/39	30398/7.07	190	41%
15	ribosomal protein L23, putative [*Eimeria tenella*]	XP_013230060.1	15/37	20662/10.50	163	49%
**Group 5**	**Protein translation**					
17	eukaryotic translation initiation factor 4e, putative [*Eimeria tenella*]	XP_013231945.1	12/42	29332/6.63	158	36%
18	zinc finger (C3HC4 RING finger) protein, putative, partial [*Eimeria tenella*]	XP_013234909.1	16/42	15947/6.77	206	63%

a IDs correspond to protein spots revealed by 2-dimensional gel electrophoresis (2-DE) (as shown in Fig. 1) and divided into five non-overlapping groups.

b Accession number as captured in NCBI.

c Number of peptides that match the predicted protein sequence.

d Protein score is −10*Log (P), where P is the probability that the observed match is a random event. Protein scores greater than 71 are considered statistically significant (*p* < 0.05).

e Percentage of predicted protein sequence covered by matched peptides.

**Table 2:** Protein amino acid sequence homology among three common *Eimeria* species of interest.

Spot ID^a^	Accession number	Amino acid sequence identity (%)^b^			SecP/SP/TM prediction^e^
		*E. tenella*	*E. necatrix* ^*c*^	*E. maxima* ^*d*^	
01	XP_013228359.1	100%	88.6%	76.6%	0.796*/N/Y
02	XP_013235934.1	100%	–	60.7%	0.402/N/N
03	XP_013229187.1	100%	97.8%	82.7%	0.198/Y/Y
04	XP_013229466.1	100%	71.1%	49.6%	0.959*/N/Y
05	XP_013233623.1	100%	96.1%	37.9%	0.421/N/N
06	XP_013229795.1	100%	96.9%	71.0%	0.493/N/N
07	XP_013230117.1	100%	96.0%	56.6%	0.075/N/N
08	XP_013231415.1	100%	–	–	0.090/N//N
09	XP_013232446.1	100%	98.8%	82.7%	0.748*/N/N
10	XP_013231991.1	100%	95.2%	–	0.711*/N/N
11	XP_013234769.1	100%	95.2%	68.0%	0.476/N/N
12	XP_013227835.1	100%	97.5%	86.4%	0.140/N/Y
13	XP_013234425.1	100%	–	–	0.674*/N/N
14	XP_013233504.1	100%	98.0%	87.5%	0.060/N/N
15	XP_013230060.1	100%	–	66.1%	0.640*/N/N
16	XP_013229897.1	100%	94.9%	–	0.120/N/N
17	XP_013231945.1	100%	–	80.0%	0.264/N/N
18	XP_013234909.1	100%	–	81.8%	0.810*/N/N

a IDs correspond to protein spots revealed by 2-dimensional gel electrophoresis (2-DE) gel spot number (as shown in Fig. 1).

b All protein sequences in the same line were aligned with *Eimeria tenella* using the BLAST tool on the NCBI online website.

c, d The protein sequences of *E. necatrix* and *E. maxima* are compared with the amino acid sequences of *E. tenella*. The (–) symbol in the table indicates that the corresponding orthologs have not been found.

e Non-classical secretory property (SecP) and presence of a signal peptide (SP) and transmembrane domains (TM) were analyzed using three servers: SecretomeP-2.0a Server (https://services.healthtech.dtu.dk/services/SecretomeP-2.0/), SignalP-5.0 Server (https://services.healthtech.dtu.dk/services/SignalP-5.0/), and TMHMM Server v. 2.0 (https://services.healthtech.dtu.dk/services/TMHMM-2.0/), respectively.

Abbreviations: *, a SecP score exceeds the threshold (0.6), indicative of a secretory potential through the non-classical pathway for proteins with no signal peptides; N, not present; Y, yes.

## Discussion

Our study extends previous work by focusing on three main *Eimeria* pathogens [21]. Using immunoproteomics techniques and mass spectrometry, our research uncovered 18 evolutionarily conserved immunogens in *E. tenella* sporozoites that are cross-reactive with hyperimmune sera raised against *E. tenella* and two other related species. These proteins were assigned to diverse and complex functions, including protein translation and transport, motility, and enzymatic activity. Of note, the amino acid sequences of four immunodominant immunogens (protein spots #03, #09, #12, and #14) share over 80% similarity among three *Eimeria* species. Among these, protein #03 is predicted to have both a signal peptide and transmembrane region, suggesting extracellular secretion or into specific organelles *via* the classical secretion pathway and possible participation in intercellular communication and related functions. In contrast, the remaining 17 proteins all lack a signal peptide, and the SecP scores of 7 proteins exceed 0.6, possibly localized in specific intracellular compartments or metabolic processes [26]. Presence of transmembrane domains in three other immunogens (proteins #01, #04, and #12) may potentially facilitate ready recognition by the immune system and offer targets for direct interactions with immune effector molecules [14].

In terms of optimal candidates for a multi-species *Eimeria* vaccine, sequence homology is of top priority, while SecP scores (*e.g.*, > 0.6) or transmembrane presence for surface accessibility and proven immunogenicity are also critical. For example, one translation initiation factor (protein #17) has been shown to induce tumor necrosis factor-alpha (TNF-α) production and inhibit the growth of *Toxoplasma gondii* [10]. One Zinc finger protein (protein #18) is known to regulate *E. tenella* gene expression, leading to reduced cecal lesions and oocyst output in infected chickens [37]. Direct targeting of these essential proteins, as well as several transmembrane candidates cited above, is worth further downstream evaluation.

Conserved immunogens with enzymatic activities include an ATP-binding cassette (ABC) protein (protein #01), an adenylate kinase (protein #09), a purine nucleoside phosphorylase (protein #11), and a vacuolar ATPase synthase subunit d (protein #14). The ABC transporter subfamily B member 2 (ABCB2) is involved in the transport of various substrates [4, 19]. Vacuolar ATPase is a highly conserved proton pump that maintains cellular energy balance and regulates organelle pH [25], while purine nucleoside phosphorylase is essential in purine metabolism [27]. The importance of these conserved immunogens is expected to go beyond vaccination, as they could also serve as therapeutic targets.

Among the motility-related proteins, a kinesin motor domain-containing protein (protein #05) has also been shown to have immune protective effects by stimulating the proliferation of peripheral mononuclear cells and the production of IgG2 antibodies [12]. The ATP-dependent metalloprotease FtsH (protein #08) degrades misfolded/excess proteins, which has been shown to be crucial for the infectivity and *in vitro* growth of *Borrelia burgdorferi*, the causative agent of Lyme disease [6]. Studies have also shown that the 26S proteasome non-ATPase regulatory subunit (PSMD, protein #16) may enhance immune cell functions [20]. Endonuclease/exonuclease/phosphatase domain-containing proteins (protein #04), as key immune regulators, participate in immune cell DNA repair, immune response modulation, and immune cell apoptosis [24, 34]. However, other proteins in this functional group have limited information to infer their potential as suitable vaccine candidates.

Overall, our study is consistent with earlier observations that conserved *Eimeria* immunogens not only exhibit multiple functions for various pathways, but also demonstrate potent antigenicity in immunoassays [13, 28]. For proteins involved in multiple essential functions, immune targeting should be suitable for different developmental stages [18, 35]. To this end, our findings here build on previous studies on *Eimeria* and related immunogens by identifying highly cross-reactive proteins in sporozoites, differing from earlier work that dealt with immunogens like cross-reactive microneme proteins from *E. acervulina*, *E. maxima*, and *E. mitis* sporozoites [35], while also extending Liu *et al.*’s analysis of conserved antigens from *E*. *acervulina*, *E*. *tenella*, and *E*. *necatrix* sporozoites [22]. The proteins with predicted enzymatic functions or involved in translation offer broad, intracellular targets for immune and other interventions, as seen with multi-epitope DNA vaccines [9], which should supplement existing strategies that focus on antibody protection induced by recombinant antigens [33] or live attenuated vaccines [23].

Of note, the hyperimmune sera used in this study may overestimate immunogenicity, and follow-up studies are needed to validate these using immune sera from naturally infected birds. Likewise, while bioinformatics predictions provide a basis for screening protein functions, they are unable to replace functional assays (*e.g.*, gene knockdowns) as the gold standard for direct verification.

## Conclusions

Immunoproteomics is an effective way of identifying conserved immunogens with broad, cross-reactivity across three *Eimeria* species of veterinary importance. Our findings can serve as a solid foundation for the design and clinical evaluation of novel, next-generation vaccines for combating mixed infections, especially if additional immunoassays (*e.g.*, ELISA and immunization trials) are used to validate the immunogenicity of individual immunogens or their subunits. Further verification using protective immune sera from naturally infected hosts against recombinant immunogens should also benefit such follow-up efforts.
